# Flavonoid epimers from custard apple leaves, a rapid screening and separation by HSCCC and their antioxidant and hypoglycaemic activities evaluation

**DOI:** 10.1038/s41598-020-65769-5

**Published:** 2020-06-01

**Authors:** Heng Zhu, Long Chen, Jinqian Yu, Li Cui, Iftikhar Ali, Xiangyun Song, Jeong Hill Park, Daijie Wang, Xiao Wang

**Affiliations:** 1 Key Laboratory of TCM Quality Control, Shandong Analysis and Test Center, Qilu University of Technology (Shandong Academy of Sciences), Jinan, 250014 China; 2 Department of Chemistry, Karakoram International University, 15100 Gilgit, Pakistan; 3 College of Pharmacy and Research Institute of Pharmaceutical Sciences, Seoul National University, Seoul, 08826 Korea; 4 College of Life Science, Shandong Normal University, Jinan, 250014 China

**Keywords:** Medicinal chemistry, Techniques and instrumentation

## Abstract

Leaves of custard apple are widely used in many places as a popular dietary supplement for the treatment of diabetes. Flavonoids are known to have anti-diabetic activity. In this study, the main flavonoid epimers were separated. The crude extract was first screened by HPLC-DAD before and after incubation with DPPH method to evaluate the antioxidants. An efficient extraction method was employed to remove non-flavonoid components. Subsequently, five main flavonoids with two pairs of epimers including quercetin-3-*O*-robinobioside, rutin, quercetin-3-*O*-*β*-D-glucoside, kaempferol-3-*O*-robinobioside, and kaempferol-3-*O*-rutinoside were successfully separated by high-speed counter-current chromatography with ethyl acetate/*n*-butanol/water (4:1:5, *v/v*) coupled with online-storage inner-recycling mode. The structures of the separated compounds were identified by spectral techniques. The purity of the separated flavonoid glycosides was over 98%, as determined by HPLC. The separated pure constituents were found to possess the antioxidant capacities following DPPH radical scavenging protocol. The compounds (**1**-**3**) exhibited better antioxidant activity. Furthermore, the glucose uptake of crude flavonoid extract had better results than the crude ethanol extract. The present study demonstrates that the efficacy of custard apple leaves in lowering glucose level, and antioxidant capacities of separated pure compounds probably appear to be predominantly responsible for hypoglycaemic properties on HepG2 cells.

## Introduction

Custard apple (*Annona squamosa* Linn.; Annonaceae family), a well-known tropical fruit for the nutritional value, is widely spread in many areas of the world including Southeast Asia, South America, the West Indies, and Central America. Leaves of custard apple are a useful folklore medicine and rich in secondary metabolites such as flavonoids, alkyl ketones, sesquiterpenes, essential oil^1–6^. However certain secondary metabolites have recently also been reported from other parts of *A. squamosa* L. including diterpenes^7^, acetogenins^8^, isoquinoline alkaloid^9^ etc. Leaves of custard apple are reported as a popular dietary supplement for the treatment of diabetes^10,11^, and as functional food and daily tea for promoting digestion, improving sleep and treating diabetes^12,13^. In addition, the leaves of custard apple are also reported for antibacterial^14,15^, anticancer^9^, antithyroidal^16^, and protective properties^17^. Thus the previous reports reveal that the custard apple leaves have good development prospect in the areas of food and medicine.

Recently, most researchers have investigated the fruit or seed part of the custard apple^18–22^. But a few researchers have focused on the antioxidant properties of custard apple leaves crude extract^23,24^. Natural antioxidant moieties are reported to control free radical chain reactions in the living body^25^. Meanwhile, oxidative stress has a significant contribution to the pathogenesis of diabetic nephropathy^26,27^. In this case, the prominent antidiabetic activity may be caused by the antioxidants present in custard apple leaves^28,29^. However, very little information is available on monomers with antioxidant and antidiabetic activities in custard apple leaves.

Traditional separation methods using silica gel, preparative HPLC or TLC for separation of complex mixtures, such as that in a crude extract of custard apple leaves, usually require multiple chromatographic steps. This leads to long separation times, consumption of large amount of solvent, a high risk of irreversible sample adsorption, and low sample recovery^30^. An effective isolation and purification technique is therefore straightway required for separation of flavonoids from custard apple leaves. For efficient separation, high-speed counter-current chromatography (HSCCC) is a good choice. It is based on liquid-liquid partition chromatography, which eliminates the possibility of irreversible sample adsorption onto the solid support. Furthermore, HSCCC gives high recovery, rapid and effective separation, is easily scaled-up, simple to operate, and has low solvent consumption. It has recently become a useful tool for preparative isolation and purification of various natural products^31–34^.

Herein, we report an efficient method to separate the flavonoid epimers from custard apple leaves. The crude flavonoids were enriched using an efficient extraction method. The structures of the isolated compounds were elucidated by ESI-MS, ^1^H- and ^13^C NMR spectra. Their antioxidant and antidiabetic activities were evaluated *in vitro* by DPPH radical scavenging and HepG2 cells respectively. This is, to the best of our understanding, the first report of the evaluation of antioxidant and antidiabetic activities with monomer compounds from custard apple leaves.

## Materials and methods

### Reagents and materials

Solvents were of analytical grade from Sinopharm Chemical Reagent Co., Ltd (Shanghai, China). Acetonitrile and acetic acid for HPLC were of chromatographic grade from Fisher Company (Fairlawn, NJ, USA). Water was purified using Milli-Q system (Millipore, Bedford, MA, USA). Fetal bovine serum (FBS), Dulbecco’s modified Eagle medium (DMEM), phosphate-buffered saline (PBS), streptomycin, and penicillin were purchased from Jinan Kang-Mai Science & Technology Co., Ltd. (Jinan, China). Glucose test kit was from Rong-Sheng Biotech Co., Ltd. (Shanghai, China).

Custard apple leaves were obtained from a Chinese traditional medicine market of Bozhou (Anhui, China) and identified by Dr. Xiao Wang (Shandong Analysis and Test Center). A voucher specimen (2017090602) was deposited in Shandong Analysis and Test Center.

### Apparatus

EMC-300 HSCCC machine (Beijing, Emilion Technology, China) with 03 separation columns of 300 mL (2.6 mm i.d.) and 20 mL manual sample loop was used. Four other instrument modules were equipped, including a TBP-5002 pump (Tauto Biotechnique, Shanghai, China), a DC-0506 water bath, a 8823A-UV Monitor (Beijing, Emilion Technology, China), and a Model 3057 portable recorder (Sichuan, Instrument Factory, China) to maintain the temperature at 25 °C. HPLC separation was performed on an Agilent 1260 HPLC system (Agilent Technologies, Santa Clara, CA, USA) consisting of a quaternary pump, an online degasser, a diode array detector, an auto-plate-sampler and a thermostatically controlled column compartment.

### Preparation of the crude extracts

The dried custard apple (*Annona squamosa* Linn.; Annonaceae family) leaves (2 kg) were refluxed with 5 L of 95% ethanol for 2 h and the procedure was repeated twice. The combined crude ethanol extract (CEE) was filtered and concentrated by rotary evaporation at 50 °C. The residue was suspended in water and extracted with petroleum ether. After removal of petroleum ether layer, the aqueous layer was extracted with *n*-butanol three times. The *n*-butanol extract was concentrated to dryness to yield 62 g of BuOH extract. The BuOH extract was distributed in petroleum ether/ethyl acetate/methanol/water (5:5:2:8, *v/v*) and the lower phase was concentrated in vacuo at 50 °C to dryness. Total 17.6 g of the crude flavonoid extract (CFE) was obtained from the custard apple leaves (0.88% yield).

### Rapid screening of antioxidant potential

Comparison of the high performance liquid chromatography (HPLC) – diode array detector (DAD) chromatogram before and after incubation with 2,2-diphenyl-1-picrylhydrazyl (DPPH) was used for the rapid screening of the antioxidant principles in the CFE. In brief, the CFE solution (0.3 mL, 2.0 mg/mL) was mixed with DPPH (0.3 mL, 30.0 mg/mL) for 30 min at 37 °C. The solution was filtered through a 0.45 μm membrane filter before HPLC analysis. HPLC peaks were compared with those of the CFE which was not treated with DPPH. Waters Symmetry C_18_ column (4.6 × 250 mm, 5 μm i.d.) as a stationary phase, and acetonitrile and 0.1% aqueous acetic acid (16:84, *v/v*) as a mobile phase were used with a flow-rate of 1.0 mL/min.

### Selection of solvent system for HSCCC

Each phase (upper and lower) of the selected solvent system was set in a tube. About 2 mg of CFE was added, and the mixture was shaken vigorously for 1 min. After the separation of two phases, 1 mL of each phase was taken and dried with nitrogen. The residue was dissolved in 1 mL of methanol which was analyzed by HPLC. The partition coefficients (*K*_D_-values) of the flavonoids were calculated using the formula *K* = *A*_S_/*A*_M_, where *A*_S_ and *A*_M_ are the peak areas of target compound in upper and lower phases, respectively.

### Preparation of solvent system and sample solutions

Two phase solvent system consisting of ethyl acetate-*n*-butanol-water (4:1:5, *v/v*) was put into a separating funnel. After shaking vigorously, the solution was left standby for few minutes and separated into two phases. The upper and lower phases were used as stationary and mobile phases respectively. To prepare the sample solution, 200 mg of the CFE was dissolved in 20 mL of isometric (1:1, upper:lower) solution.

### Online-storage inner-recycling mode

A separate external column loop also called recycling loop, on one side through the pump, on the other side through the detector, was connected with the CCC column using 6-way valves. When the target fractions, that contain more than a single compound, were required to store, coming out of the CCC column, the separation mode was changed to the recycling loop, through 6-way valve. When the fraction containing compounds mixture was stored in the recycling loop, then the separation mode was changed again to the collection mode, using 6-way valve, to elute the other separated compounds. When all the remaining fractions were eluted and collected, then the target fraction stored in the recycling loop was allowed to enter the CCC column again, through the pump. If the separation observed after recycling through the detector was found clear, the sample fractions were collected. In case if more separation was required, the fraction was recycled directly using the 6-way valve, without online-storage. The separation was continued again, thus recycled. To get more clear separation, the recycling was continued. This type of setup is called as online-storage inner-recycling mode.

### HSCCC separation procedure

In the first step, the CCC column was completely filled with the upper phase at 20 mL/min in head-to-tail elution mode. Then, the lower phase was pumped at 2.0 mL/min, while the apparatus was set at 800 rpm in clockwise, and other parameters were set; a temperature of 25 °C, and a detector wavelength 254 nm. After equilibrium reached, the sample solution was injected into the sample loop. The equilibrium was determined by the retention of the stationary phase (*S*_f_). *S*_f_ is the stationary phase relative to the total CCC column capacity, and it is calculated by *S*_f_ (%) = (*V*_T_ – *V*_E_)/*V*_T_ × 100%, where *V*_T_ is the total CCC column capacity (usually 320 mL), and *V*_E_ is the volume of the stationary phase eluted from the CCC column loop (120 mL observed in our present study). As a result, the calculated *S*_f_ was found 62.5% in our present experiment. When the sample eluent for the inner-recycling separation was at the tail end of the CCC, the separation mode was changed to online-storage mode. After the target fractions were collected in the storage-loop, the 6-way valve was turned to collection mode for further separation. When the target fractions were eluted, the CCC instrument was stopped and the solvent was blown out with nitrogen. The new solvent system was re-equilibrated for the secondary separation of the collected fractions.

### HPLC analysis and structural identification

HPLC analyses of the samples were performed on Agilent 1260 HPLC (analytical) equipment with a Waters symmetry C_18_ column. A mixture of acetonitrile and 0.1% aqueous acetic acid (16:84, *v/v*) was chosen as the mobile phase with a flow-rate of 1.0 mL/min and a wavelength of 254 nm. The separated compounds were identified by ESI-MS, ^1^H and ^13^C NMR spectra. The ESI-MS (positive ion mode, and negative ion mode) spectral analysis was carried out on Agilent 6520 Q-TOF (Agilent, Santa Clara, CA, USA). The NMR spectra were recorded on a Bruker AV-400 spectrometer (Bruker BioSpin, Rheinstetten, Germany) with DMSO as solvent and chemical shift (*δ*) values were expressed in parts per million (ppm), and the coupling constant (*J*) in Hz.

### Evaluation of antioxidant activity of the pure compounds

The antioxidant capacity was determined by DPPH assay with appropriate modification^35^. In brief, DPPH (2.5 mg) was dissolved in 100 mL ethanol to make a standard solution at a concentration of 25 μg/mL. Serial dilution was used to provide standard solutions at concentrations of 0, 5, 10, 15, 20, and 25 μg/mL in ethanol. The absorbance values of the six solutions were measured by ultraviolet spectrophotometry at 517 nm to prepare the standard curve. DPPH (2.0 mg) was dissolved in 100 mL ethanol to make a standard solution at a concentration of 20 μg/mL. Solutions containing different concentrations of *L*-ascorbic acid (positive control), the CFE and monomeric compounds were prepared as test samples. Briefly, 3 mL of standard solution was poured to a 10 mL colorimetric tube together with 2 mL of the solution from the sample group. For the control group, 3 mL of ethanol was added to a 10 mL colorimetric tube together with 2 mL of the sample solution, while 3 mL of the standard solution with 2 mL of ethanol was used as the blank group. The absorbance at 517 nm was determined by ultraviolet spectrophotometry for each mixture incubated for 30 min at 37 °C. Each concentration was tested thrice and the results were calculated as the average value. The antioxidant potential was calculated as the percentage of DPPH radical elimination as follows:

Scavenging rate (%) = [*A*_blank_ − (*A*_sample_ − *A*_control_)]/*A*_blank_ × 100%

where *A*_blank_, *A*_control_ and *A*_sample_ were the absorbance values of blank, control and sample solutions at different concentrations, respectively.

### Hypoglycaemic activity *in vitro*

#### Cytotoxicity assay on HepG2 cells

The cytotoxic activity of the CEE, the CFE and the monomeric compounds was determined in HepG2 (a human hepatoma cell line) cells by the MTT assay^36^. HepG2 cells were cultured in DMEM supplemented with penicillin (100 U/mL)/streptomycin (100 μg/mL) and 10% FBS. The cells were incubated at 37 °C and 5% CO_2_. Trypsin solution was used to digest HepG2 cells in the logarithmic growth phase. The cell density was then adjusted to 5 × 10^4^ /mL with the culture medium. The cells were seeded in a 96-well cell culture plate at the volume of 100 μL/well at 37 °C and 5% CO_2_. The seeded cells were treated with the CEE, CFE, and the separated pure compounds at appropriate concentrations for 24 h, respectively. The wavelength of the absorbance was 570 nm to determine the cell viability. The effect of the components on cell viability was calculated as the following formula: Cell viability (%) = *A*_570nm_ of the treated sample / *A*_570nm_ of the untreated sample × 100%.

#### Glucose consumption assay on HepG2 Cells

100 μL of the cell suspension (5 × 10^4^ /mL) was seeded to the 96-well cell culture plate and cultured at 37 °C and 5% CO_2_. After the cells were cultured for 24 h, the old medium was aspirated and the wells were washed with PBS solution twice, then serum-free DMEM medium with insulin solution was added synchronously to the cells. The supernatant was aspirated after cultured for 36 h and the cells were poured with serum-free drug-containing or drug-free DMEM added. The experiment was divided into four groups: components treatment group (30-120 μg/mL), blank control group, Metformin (Met) group (30-120 μg/mL), and insulin group (10^-5^ mmol/L). The glucose content was detected at 505 nm after 24 h of culture according to the glucose test kit (Beijing Applygen Technologies Inc., China). The glucose consumption rate was calculated with the following formula: ∆GC = (glucose concentration of blank wells - glucose concentration of cell cell-inoculated).

#### Statistical analysis

All results are expressed as means ± SD. Statistical significance was tested by one-way analysis of variance using *SPSS* software. *p*-Values of less than 0.05 were considered statistically significant.

## Results and discussion

### HPLC-DAD before and after incubation with DPPH analysis

**Figure 1:**
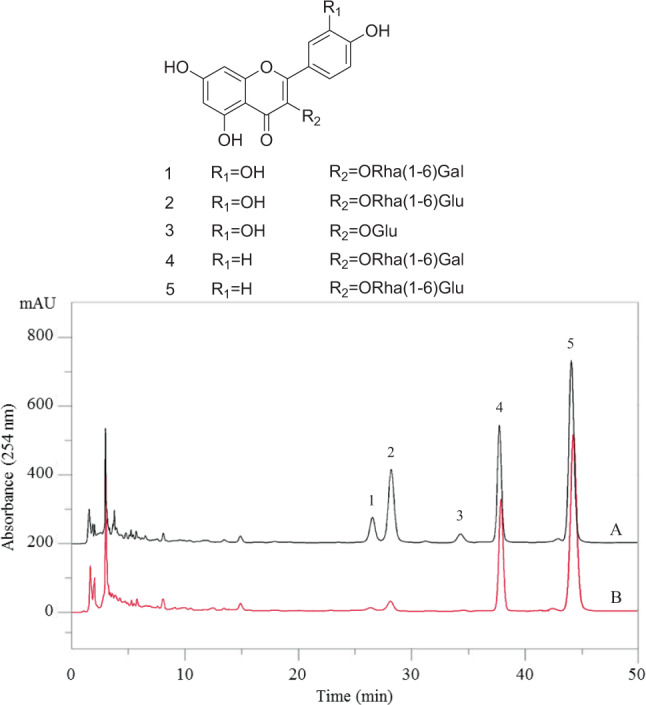
Chemical structures of the separated flavonoids and DPPH based HPLC chromatograms of the crude flavonoid extract (CFE) from the custard apple leaves. Experimental conditions: a Waters Symmetry C_18_ column (4.6 × 250 mm, 5 μm i.d.); Mobile phase: acetonitrile/0.1% acetic acid solution (16:84, *v*/*v*). (**A**) Before reaction, (**B**) after reaction.

The HPLC-DAD before and after incubation with DPPH is a rapid screening method to understand the antioxidant capacity which is evaluated by the decrease of peak areas in the HPLC chromatogram after reaction with DPPH. The peak areas of compounds with strong antioxidant capacity would be decreased, while those of non-antioxidants would be unchanged^37^. Figure 1A,B shows the effect of the CFE after reaction with DPPH. As shown in Fig. 1B, the decrease in peak area is very different, with peak 1 and 2 significantly reduced. Peak 3 in Fig. 1 disappeared after reaction. This indicated that the main compounds of peaks 1, 2 and 3 in the CEE of custard apple leaves possess higher antioxidant properties. Later on, the DPPH radical scavenging activity was determined for the separated pure compounds.

### Removal of non-flavonoid components

Plant leaves are an important source of natural products and rich in medicinal components. However, plant leaves also contain large amount of chlorophyll, wax, and other components that can impact separation. Traditionally, the removal has been conducted by extraction with low-polarity solvent such as petroleum ether. This method is only partially successful and moderately polar components remain non-separated. Herein, an efficient method was used to remove non-flavonoid components from custard apple leaves according to the *K*_D_-values of the target compounds. As shown in Table 1, the *K*_D_-values of compounds 1–5 were far lower than in the *n*-hexane/ethyl acetate/methanol/water (5:5:2:8, *v/v*) two-phase solvent system. This indicated that the flavonoids in custard apple leaves were mainly distributed into the lower hydrophilic solvent. The polarity of the upper phase (*n*-hexane and ethyl acetate) is higher than that of petroleum ether. More non-flavonoid components were removed using this method compared with petroleum ether extraction.

**Table 1:** The *K*_D_-values of compound peaks in HSCCC separation with different solvent systems.

Solvent system	*K*_D_*-*values of the isolated compounds				
	**1**	**2**	**3**	**4**	**5**
CHCl_3_/MeOH/H_2_O (4:3:3, *v/v*)	5.18	7.65	6.49	14.36	12.45
*n*-hexane/EtOAc/MeOH/H_2_O (5:5:2:8, *v/v*)	<<1	<<1	<<1	<<1	<<1
EtOAc/H_2_O (1:1, *v/v*)	0.12	0.19	0.58	0.14	0.18
EtOAc/*n*-BuOH/H_2_O (4.5:0.5:5, *v/v*)	0.66	0.74	3.28	0.77	1.04
EtOAc/*n*-BuOH/H_2_O (4:1:5, *v/v*)	1.76	2.03	5.35	2.47	3.18

**Figure 2:**
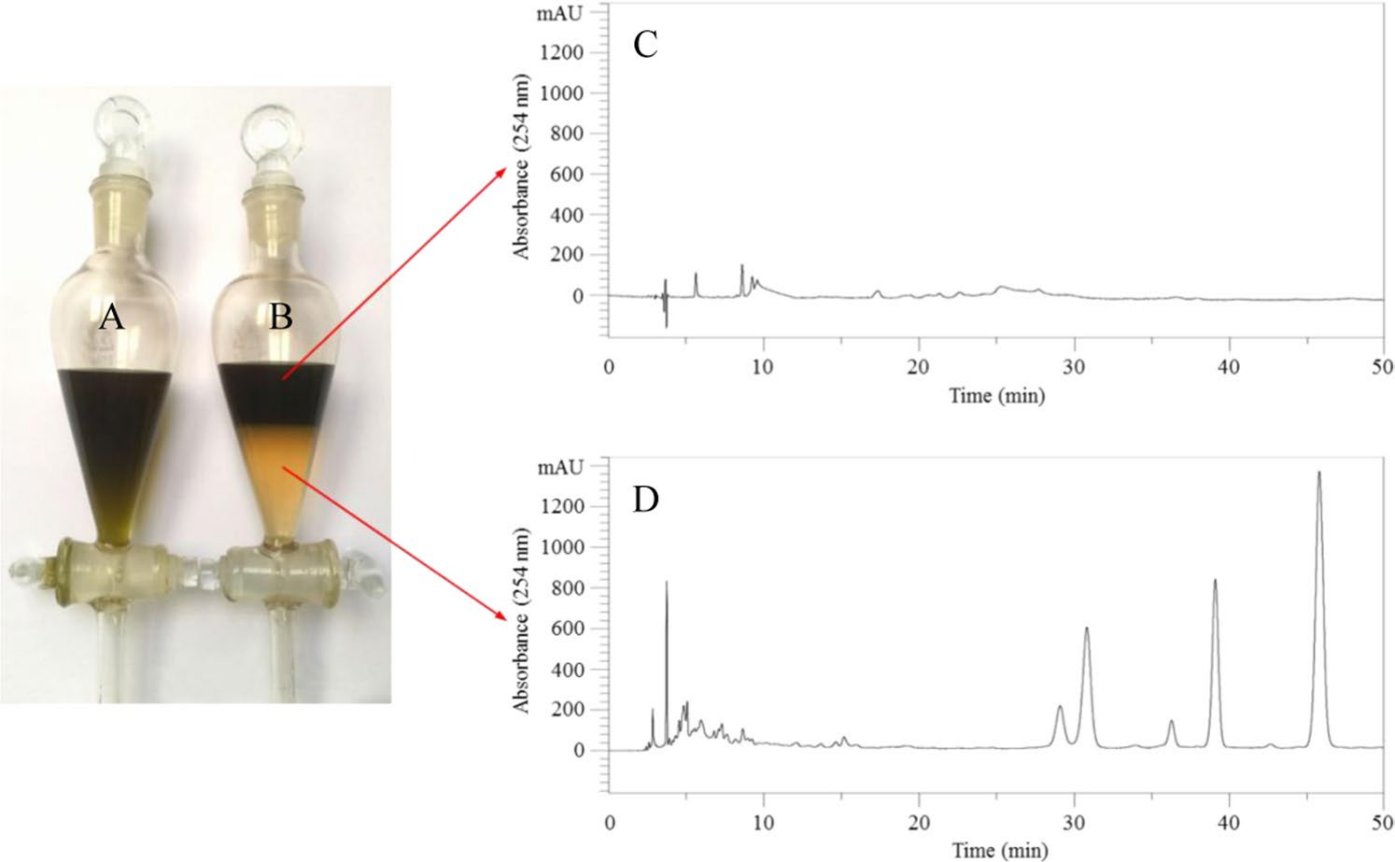
The images and chromatograms showing the procedure for removal of chlorophyll and wax from the *n*-butanol extract of custard apple leaves. (**A**) *n*-butanol extract, (**B**) the fractionation of *n*-butanol extract with *n*-hexane/ethyl acetate/methanol/water (5:5:2:8, *v*/*v*), **(C**) HPLC chromatogram of the upper phase after distribution, (**D**) HPLC chromatogram of the lower phase after distribution.

The *n*-butanol extract from the custard apple leaves in aqueous solution (Fig. 2A), and the distribution of *n*-butanol extract in *n*-hexane/ethyl acetate/methanol/water (5:5:2:8, *v/v*) (Fig. 2B) are shown in respective figures. Comparing the two figures, the chlorophyll, wax and other components were mainly distributed in the deeply colored upper phase (Fig. 2B). The analytical HPLC chromatograms of the upper and lower phases are shown in Fig. 2C,D respectively. It is apparent that the upper phase contained almost no flavonoids after extraction with the corresponding lower phase. The CFE thus obtained was 17.609 g, *i.e*. the yield coefficient was 28.4% (*w*/*w*) from 62 g of the *n*-butanol extract.

### HSCCC solvent system selection and separation

Several mixed-solvent systems were tested, including chloroform/methanol/water (4:3:3, *v*/*v*), *n*-hexane/ethyl acetate/methanol/water (5:5:2:8, *v/v*), ethyl acetate/water (1:1, *v/v*), and ethyl acetate/*n*-butanol/water (4.5:0.5:5, 4:1:5, *v*/*v*). When chloroform/methanol/water (4:3:3, *v*/*v*) was used, the flavonoids were mainly distributed in the upper phase, with *K*_D_ values far greater than 1, making them difficult to elute. When *n*-hexane/ethyl acetate/methanol/water (5:5:2:8, *v/v*) solvent system was used, the flavonoids were mainly found in the lower phase, such that the target compounds would be rapidly eluted with no peak resolution. The ethyl acetate/*n*-butanol/water solvent system was then tested. As shown in Table 1, the *K*_D_ values increased as the proportion of *n*-butanol was increased. The *K*_D_ values in ethyl acetate/water (1:1, *v/v*) were rather low. When using ethyl acetate/*n*-butanol/water (4.5:0.5:5, *v/v*), appropriate *K*_D_ values, in the range of 0.66–3.28, were obtained. However, the *α* values of *K*_2_/*K*_1_ and *K*_4_/*K*_2_ were 1.12 and 1.04, indicating that these three compounds would be difficult to separate. Changing the solvent system to ethyl acetate/*n*-butanol/water (4:1:5, *v/v*) gave higher *K*_D_ values, in the range of 1.76–5.35. The *α* values of *K*_2_/*K*_1_ and *K*_4_/*K*_2_ were 1.15 and 1.22, respectively. In view of the *K*_2_/*K*_1_ *α* value and the *K*_D_ value of compound 5, a combination of blowout and inner-recycling mode was used for further HSCCC separation.

**Figure 3:**
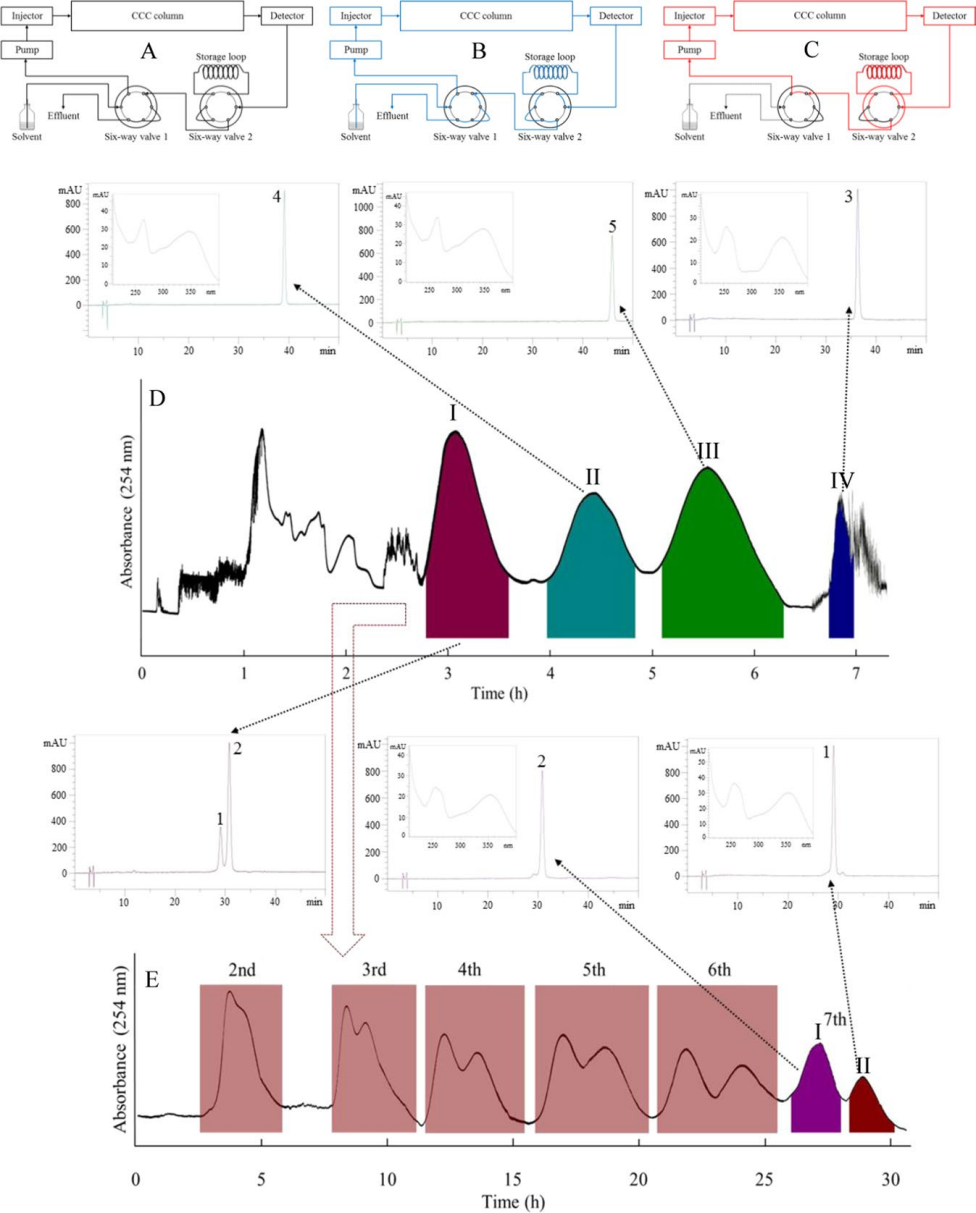
Online-storage inner-recycling CCC procedure on separation of flavonoids from custard apple leaves. Solvent system: ethyl acetate-*n*-butanol-water (4:1:5, *v/v*); Flow-rate: 2.0 mL/min, Detection wavelength: 254 nm. (**A–C**) Schematic diagrams of the online-storage inner-cycle CCC separation, (**A**) collection mode, (**B**) online-storage mode, (**C**) inner-recycling mode), (**D**) HSCCC chromatogram of 1D separation mode, (**E**) HSCCC chromatogram of online-storage inner-recycling CCC separation mode on separation Fr. I in Fig. 4B.

**Figure 4:**
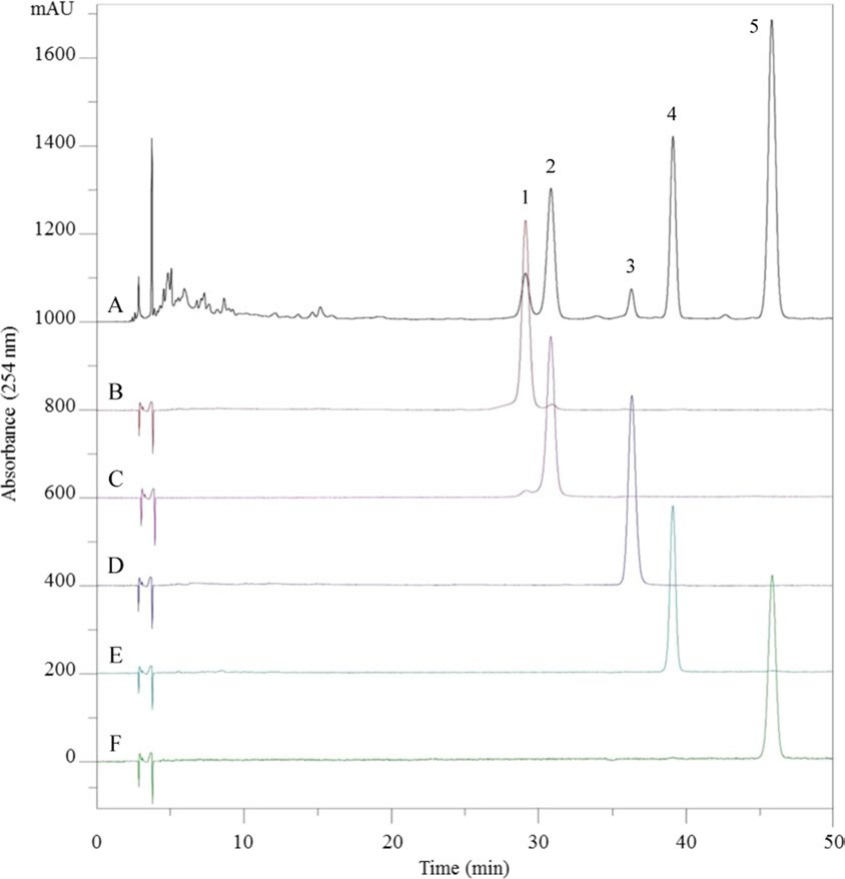
HPLC chromatograms of the crude flavonoid extract (CFE) and the isolated flavonoids. Experimental conditions: Waters Symmetry C_18_ column (5 μm, 4.6 mm × 250 mm, i.d.,); Mobile phase: acetonitrile/0.1% aqueous solution of acetic acid (16:84, *v/v*). Flow rate: 1.0 mL/min; Detection: 254 nm. (**A**) crude extract, (**B**) peak II in Fig. 3E, (**C**) peak I in Fig. 3E, (**D**) peak IV in Fig. 3D, (**E**) peak II in Fig. 3D, (**F**) peak III in Fig. 3D.

The number of theoretical plates is correlated to the length of the column and can be expressed by the equation (*R*_1_/*R*_2_)^2^ = *N*_1_/*N*_2_ = *L*_1_/*L*_2_, where *R* is the resolution, *N* is the total number of theoretical plates, and *L* is the length of the column^38^. The schematic diagrams of the online-storage inner-recycling CCC mode corresponding to collection, online-storage, and inner-recycling stages are shown in Fig. 3A–C. Firstly, the CFE was eluted in collection mode (Fig. 3D). After the front of peak I was reached in HSCCC, the separation mode was switched to online-storage and peak I, containing compounds **1** and **2**, was introduced to the storage loop. After complete collection, the separation mode was switched to collection. Peaks II and III, containing compounds **4** and **5**, were eluted and collected. Since the *K*_D_ value of compound **5** (in peak IV) was 5.35, it was obtained by blowout mode to shorten the separation time. From 200 mg of the CFE, compounds **4** (peak II in Fig. 3D), 5 (peak III in Fig. 3D) and 3 (peak IV in Fig. 3D) were obtained, yielding 24, 49 and 5 mg, respectively, with over 98% purity as determined by analytical HPLC (Fig. 4). Peak I was then separated by inner-recycling mode. Good separation was achieved after six cycles. Compounds **1** (9 mg) and **2** (23 mg) were obtained with purity greater than 98% as determined by HPLC (Fig. 4). Recently, the compounds **1**–**5** have been reported by our group^39^ following linear gradient coupled with inner-recycling mode, and these compounds have also been reported from *Annona coriacea* Mart^40^. The present study demonstrates the online-storage inner-recycling HSCCC mode for the separation of the target compounds.

### Structure identification

*Compound 1 (Peak II in* Fig. 3E*)*: yellow solid (MeOH), ESI-MS (negative ion mode) *m/z*: 609.1474. ^1^H-NMR (DMSO-*d*_6_, 400 MHz) *δ*_H_: 12.57 (1 H, brs, 5-OH), 7.64 (1 H, dd, *J* = 8.50, 2.20 Hz, H-6′), 7.53 (1 H, d, *J* = 2.10 Hz, H-2′), 6.81 (1 H, d, *J* = 8.43 Hz, H-5′), 6.39 (1 H, d, *J* = 1.20 Hz, H-8), 6.19 (1 H, d, *J* = 1.20 Hz, H-6), 5.32 (1 H, d, *J* = 7.59 Hz, H-1″), 4.40 (1 H, brs, H-1′′′), 1.06 (3 H, d, *J* = 6.38 Hz, H-6′′′). ^13^C-NMR (DMSO-*d*_6_, 100 MHz) *δ*_C_: see Table 2. Thus, the structure of **1** was defined as quercetin-3-*O*-robinobioside by comparison of its MS, ^1^H and ^13^C NMR data with literature^39,41^.

**Table 2:** ^13^C NMR data of compounds (1-5) in DMSO-*d*_6_ (*δ* in ppm).

Carbon	**1** (*δ*_C_)	**2** (*δ*_C_)	**3** (*δ*_C_)	**4** (*δ*_C_)	**5** (*δ*_C_)
2	156.3	156.3	156.3	156. 5	156.6
3	133.4	133.1	133.2	133. 4	133.2
4	177.3	177.2	177.3	177. 5	177.5
5	161.2	161.1	161.1	161. 3	161.2
6	98.7	98.5	98.9	98. 8	98.7
7	164.1	163.8	163.9	164.3	164.2
8	93.5	93.5	93.8	93. 8	93.7
9	156.4	156.5	156.0	156. 7	156.8
10	104.0	103.9	104.0	104. 0	103.9
1′	121.0	121.0	121.0	121.0	120.8
2′	115.1	115.1	115.2	131.0	130.8
3′	144.9	144.6	144.9	115. 1	115.1
4′	148.4	148.3	148.9	160. 1	160.0
5′	116.0	116.1	116.1	115. 1	115.1
6′	122.0	121.5	121.8	131.0	131.0
1″	102.0	101.0	101.0	102. 1	101.3
2″	71.1	73.9	74.0	71. 2	74.2
3″	73.1	76.3	76.8	73.8	76.4
4″	68.2	70.2	70.0	68. 5	70.0
5″	73.5	75.7	77.9	73. 3	75.8
6″	65.0	66.9	61.0	65. 6	67.0
1′′′	100.0	100.6		100. 3	100.8
2′′′	70.4	70.0		70. 7	70.3
3′′′	70.7	70.4		70.8	70.6
4′′′	71.9	71.7		72.2	71.8
5′′′	68.1	68.1		68. 3	68.3
6′′′	18.0	17.8		17.9	17.7

*Compound 2 (Peak I in* Fig. 3E*)*: yellow solid (MeOH), ESI-MS (negative ion mode) *m/z*: 609.1475. ^1^H-NMR (DMSO-*d*_6,_ 400 MHz) *δ*_H_: 12.59 (1 H, brs), 7.54 (1 H, dd, *J* = 8.0, 2.0 Hz, H-6′), 7.51 (1 H, d, *J* = 1.9 Hz, H-2′), 6.90 (1 H, d, *J* = 8.20 Hz, H-5′), 6.40 (1 H, d, *J* = 1.51 Hz, H-8), 6.19 (1 H, d, *J* = 1.52 Hz, H-6), 5.34 (1 H, d, *J* = 8.04 Hz, H-1″), 4.40 (1 H, brs, H-1′′′), 1.14 (3 H, d, *J* = 6.01 Hz, H-6′′′). ^13^C-NMR (DMSO-*d*_6_, 100 MHz) *δ*_C_: see Table 2. Thus, the structure of **2** was defined as rutin and the experimental data was found similar as compared to the literature data^39,42^.

*Compound 3 (Peak IV in* Fig. 3D*)*: yellow solid (MeOH), ESI-MS (positive ion mode) *m/z*: 465.0780. ^1^H-NMR (DMSO-*d*_6,_ 400 MHz) *δ*_H_: 12.64 (1 H, s), 7.70 (1 H, d, *J* = 8.51 Hz, H-6′), 7.57 (1 H, brs, H-2′), 6.32 (1 H, d, *J* = 1.52 Hz, H-8), 6.83 (1 H, d, *J* = 8.52 Hz, H-5′), 6.20 (1 H, d, *J* = 1.51 Hz, H-6), 5.46 (1 H, d, *J* = 7.01 Hz, H-1″). ^13^C-NMR (DMSO-*d*_6_, 100 MHz) *δ*_C_: see Table 2. Thus, the structure of **3** was defined as quercetin-3-*O*-*β*-D-glucoside and its MS, ^1^H and ^13^C NMR data resembled with literature data^39,43^.

*Compound 4 (Peak II in* Fig. 3D*)*: yellow crystalline solid (MeOH), ESI-MS (positive ion mode) *m/z*: 595.1671. ^1^H-NMR (DMSO-*d*_6,_ 400 MHz) *δ*_H_: 12.57 (1 H, s), 10.20 (1 H, brs), 8.04 (2 H, d, *J* = 8.91 Hz, H-2′, H-6′), 6.87 (2 H, d, *J* = 8.89 Hz, H-5′, H-3′), 6.41 (1 H, d, *J* = 2.01 Hz, H-8), 6.22 (1 H, d, *J* = 2.05 Hz, H-6), 5.33 (1 H, d, *J* = 7.61 Hz, H-1″), 4.40 (1 H, s, H-1′′′), 1.07 (3 H, d, *J* = 6.24 Hz, H-6′′′). ^13^C-NMR (DMSO-*d*_6_, 100 MHz) *δ*_C_: see Table 2. Thus, the structure of **4** was defined as kaempferol-3-*O*-robinobioside by comparison of its MS, ^1^H and ^13^C NMR data with literature data^39,44^.

*Compound 5 (Peak III in* Fig. 3D*)*: yellow solid (MeOH), ESI-MS (positive ion mode) *m/z*: 595.1694. ^1^H-NMR (DMSO-*d*_6,_ 400 MHz) *δ*_H_: 12.55 (1 H, s, 5-OH), 10.09 (1 H, s, 4′-OH), 7.97 (2 H, dd, *J* = 8.82 Hz, H-2′, H-6′), 6.86 (2 H, dd, *J* = 8.81 Hz, H-3′, H-5′), 6.40 (1 H, d, *J* = 2.11 Hz, H-8), 6.35 (1 H, d, *J* = 2.01 Hz, H-6), 5.29 (1 H, d, *J* = 7.21 Hz, H-1″), 4.36 (1 H, brs, H-1′′′), 0.97 (3 H, d, J = 6.04 Hz, H-6′′′). ^13^C-NMR (DMSO-*d*_6_, 100 MHz) *δ*_C_: see Table 2. Thus, the structure of **5** was defined as kaempferol-3-*O*-rutinoside by comparison of its MS, ^1^H and ^13^C NMR data with literature data^39,45^.

### Antioxidant activity of the isolated compounds

Flavonoids are well-known antioxidant components of plant species. The antioxidant activity of flavonoids is dependent on the degree and position of hydroxylation of the parent nucleus^46^. Radical scavenging activities of crude extracts from custard apple leaves have been investigated and found to have high antioxidant activity^24^.

As shown in Table 3, the antioxidant activity on % DPPH radical scavenging of the CEE, the CFE, and the isolated pure compounds were found to be in the order of quercetin-3-*O*-*β*-D-glucoside (**3**)> CFE > CEE > quercetin-3-*O*-robinobioside (**1**)> rutin (**2**)> kaempferol-3-*O*-rutinoside (**5**)> kaempferol-3-*O*-robinobioside (**4**). Quercetin-3-*O*-*β*-D-glucoside (**3**), with an IC_50_ value 69.13 ± 2.03 μg/mL, had the highest antioxidant capacity. Kaempferol-3-*O*-robinobioside (**4**) and kaempferol-3-*O*-rutinoside (**5**) showed relatively weak antioxidant capacity, with IC_50_ values of 191.67 ± 5.09 and 188.59 ± 4.14 μg/mL respectively. As depicted in Table 4, the molar concentrations (μM) of pure compounds **1** and **2** remained similar in test sample concentrations. The same way, it remained similar in case of compound **4** and **5**. This was due to the similar molecular weight and basic concentration (1 mg/mL). However, the compound **3** showed higher molar concentration in all tested samples. Thus, the maximum concentration of the compound **3** might be one reason to show remarkable activity in the experiments. However, as mentioned before, the CFE has exhibited more activity as compared to the CEE and pure compounds *i.e*. **1**, **2**, **4**, and **5**. The CFE contains all the pure components in combination, along with some molecules other than the five separated in our experiment. The molar concentration of pure compounds **1**-**5** are mentioned in Table 4.

**Table 3:** Antioxidant activity and linear range of different concentrations.

Samples	Linear range (μg/mL) ^a^	DPPH (IC_50_, μg/mL) ^a^
Crude ethanol extract (CEE)	14.62-467.84	142.50 ± 4.62
Crude flavonoid extract (CFE)	14.62-467.84	94.23 ± 2.23
Quercetin-3-*O*-robinobioside (**1**)	11.73-375.36	99.06 ± 2.32
Rutin (**2**)	20.33-325.28	105.44 ± 2.61
Quercetin-3-*O*-*β*-D-glucoside (**3**)	9.31-297.92	69.13 ± 2.03
Kaempferol-3-*O*-robinobioside (**4**)	29.34-469.44	191.67 ± 5.09
Kaempferol-3-*O*-rutinoside (**5**)	29.34-469.44	188.59 ± 4.14
*L*-Ascorbic acid ^b^	1.63-26.08	12.92 ± 0.32

^a^Each value is presented as mean ± SD (n = 3); ^b^Used as positive control.

**Table 4:** Molar concentration of pure compounds (1–5) in test samples.

Compound	Molar concentration in test samples		
	30 μg/mL	60 μg/mL	120 μg/mL
Quercetin-3-*O*-robinobioside (**1**) or Rutin (**2**)	4.91 × 10^−5^	9.83 × 10^−5^	19.67 × 10^−5^
Quercetin-3-*O*-*β*-D-glucoside (**3**)	6.47 × 10^−5^	12.93 × 10^−5^	25.86 × 10^−5^
Kaempferol-3-*O*-robinobioside (**4**) or Kaempferol-3-*O*-rutinoside (**5**)	5.05 × 10^−5^	10.10 × 10^−5^	20.20 × 10^−5^

The results, as explained in Table 3, and Table 4, suggested that antioxidant capacity was enhanced due to the presence of the hydroxyl group at the 3’ position and a smaller number of sugar bases. This mechanism has already been established previously^46,47^.

*Hypoglycaemic activities in vitro*. Table 5 shows the effect of components on cell viability with different concentrations. It was found that the tested concentrations 30-120 μg/mL had little effects on cell viability. It indicated that the components from *A. squamosa* had good safety on the HepG2 cells at high concentrations.

**Table 5:** The cell viability of different components on HepG2 cells.

Samples	Cell survival rate (%)^a^		
	30 μg/mL	60 μg/mL	120 μg/mL
Crude ethanol extract	109.24 ± 2.89	108.33 ± 3.81	111.41 ± 2.82
Crude flavonoid extract	104.91 ± 3.49	105.25 ± 4.10	104.38 ± 5.38
Quercetin-3-*O*-robinobioside (**1**)	99.48 ± 5.87	108.56 ± 6.03	110.89 ± 2.50
Rutin (**2**)	97.62 ± 6.31	91.67 ± 7.12	98.81 ± 5.66
Quercetin-3-*O*-*β*-D-glucoside (**3**)	109.20 ± 2.90	118.32 ± 3.81	111.42 ± 2.82
Kaempferol-3-*O*-robinobioside (**4**)	97.81 ± 6.19	102.03 ± 4.39	103.68 ± 2.72
Kaempferol-3-*O*-rutinoside (**5**)	108.32 ± 6.29	108.10 ± 10.72	104.41 ± 4.03
Metformin	100.16 ± 3.53	102.63 ± 3.53	100.24 ± 3.24
Blank control^b^	100		

**Figure 5:**
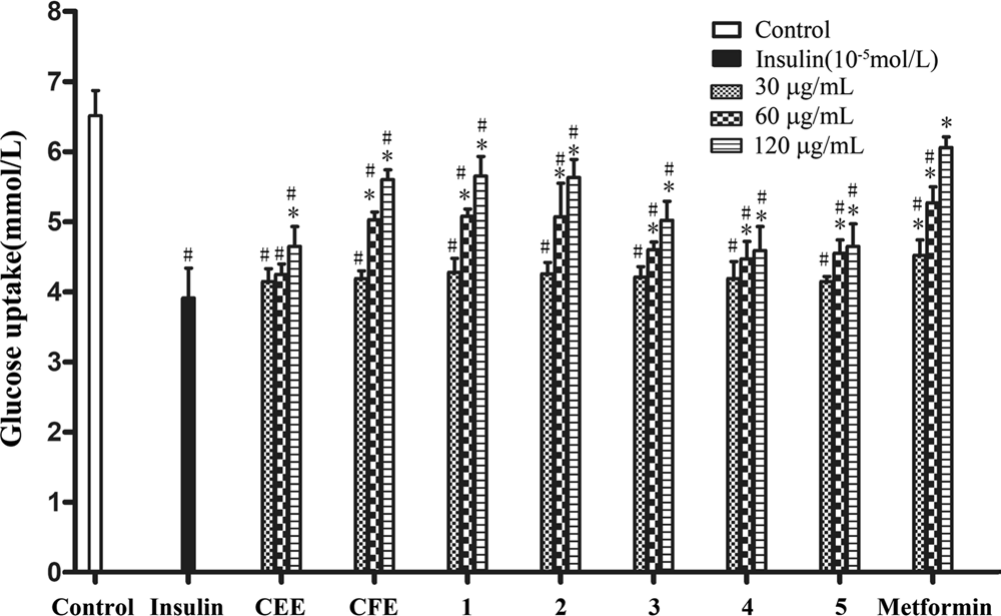
The impact of different concentrations of components on glucose consumption in insulin resistance cell model ($\bar{{\rm{x}}}$ ± s, n = 6).

Metformin is a common hypoglycaemic drug and used as the positive control. Compared with the insulin group (model groups), the CEE, the CFE and the pure compounds significantly increased the glucose uptake of HepG2 cells (Fig. 5). The data is provided in Supplementary Table.

At each concentration of the different test samples, a statistical evaluation of the data has been determined. As shown in Fig. 5, the CEE and CFE extracts showed an increased uptake of glucose in a dose-dependent manner. The effect of CFE was more significant as compared to the CEE except the low dose considering the total flavonoid content in composition more in CFE than CEE (see Supplementary Figure). Then the CFE was analyzed and main pure flavonoids were isolated. Furthermore, the pure flavonoids were able to positively affect the hepatic cells showing a dose response effect. Especially the compounds **1** and **2** had a good hypoglycaemic effect than others, but weaker than metformin. We can also speculate the different glucoside linkages and its position show different effects on its activities.

Since flavonoids have been reported to follow different pathways like direct radical scavenging, interfering with inducible nitric oxides, xanthine oxidase pathway, interaction with other enzyme system etc^47,48^. In our experiments, as the concentration of components was increased, the tendency on the glucose uptake rate of HepG2 cells increased. It indicated that the tested components had potential hypoglycaemic activity and among the flavonoid glycosides, rutin showed the highest increase of glucose uptake. Glycosylation seemed to play an important role in the uptake of glucose. Moreover, the glucose uptake of the CFE had better hypoglycaemic activity than the CEE. The removal of the non-flavonoid components may play a role of enrichment and was helpful to improve hypoglycaemic activity. Compared with the antioxidant activity, the compounds **1**-**3** had better hypoglycaemic activity than compounds **4** and **5**. Presence of a hydroxyl group at the 3’ position may enhance the hypoglycaemic activity of the flavonoids. There is significant oxidative stress in the case of diabetes, and antioxidant activity is one of the mechanisms of chronic complications of diabetes^49^. It may be concluded that probably, due to the potent antioxidant activity, the flavonoids exerted remarkable hypoglycaemic activity. The mechanism between antioxidant and hypoglycaemic activity requires further research.

## Conclusions

This study presents an efficient strategy based on HPLC-DAD before and after incubation with DPPH analysis, liquid-liquid extraction, and high-speed counter-current chromatography separation for screening and preparative separation of flavonoid epimers from custard apple leaves. The main flavonoid epimers were separated with evaluation of their antioxidant and hypoglycaemic activities *in vitro*. The extraction method, according to the *K*_D_-values of the target compounds, was efficient for removal of non-target components from the complex mixture. The use of inner-recycling CCC mode was efficient for separation of compounds with similar *K*_D_-values, reducing organic solvent consumption, enhancing the number of theoretical plates and improving peak resolution. Flavonoids with a hydroxyl group at the 3’ position had better antioxidant and hypoglycaemic activities. The established strategy for screening, enrichment and separation of hypoglycaemic component could be further used for flavonoids and may also be investigated with other natural products.

## Supplementary information

Supplementary information.
