# Supplementary material for: Flavonoid epimers from custard apple leaves, a rapid screening and separation by HSCCC and their antioxidant and hypoglycaemic activities evaluation
Source: Sci Rep. 2020 Jun 1;10:8819. doi: 10.1038/s41598-020-65769-5 (PMC7264225; doi:10.1038/s41598-020-65769-5)

-Supplementary Information-

Flavonoid epimers from custard apple leaves, a rapid screening and separation by HSCCC and their antioxidant and hypoglycaemic activities evaluation

Heng Zhu[1,+], Long Chen[1,+], Jinqian Yu[1], Li Cui[1], Iftikhar Ali[1,2], Xiangyun Song[1], Jeong Hill Park[3], Daijie Wang[1,4*] and Xiao Wang[1]

[1]Key Laboratory of TCM Quality Control, Shandong Analysis and Test Center, Qilu University of Technology (Shandong Academy of Sciences), Jinan 250014, China

[2]Department of Chemistry, Karakoram International University, 15100-Gilgit, Pakistan

[3]College of Pharmacy and Research Institute of Pharmaceutical Sciences, Seoul National University, Seoul 08826, Korea

[4]College of Life Science, Shandong Normal University, Jinan 250014, China

**Corresponding: Daijie Wang, E-Mail: wangdaijie@126.com*

Supplementary Table The different concentrations impact of components on HepG2 cells glucose uptake

Samples	ΔGC/MTT [a]		
	30 μg/mL	60 μg/mL	120 μg/mL
Crude ethanol extract	4.15±0.18#	4.45±0.15#	4.65±0.29#*
Crude flavonoid extract	4.19±0.15#	5.03±0.11#*	5.67±0.14#*
Quercetin-3-*O*-robinobioside (**1**)	4.28±0.20#	5.08±0.10#*	5.65±0.28#*
Rutin (**2**)	4.26±0.16#	5.07±0.48#*	5.63±0.26#*
Quercetin-3-*O*-*β*-D-glucoside (**3**)	4.21±0.15#	4.60±0.11#*	5.02±0.27#*
Kaempferol-3-*O*-robinobioside (**4**)	4.19±0.64#	4.47±0.28#*	4.59±0.34#*
Kaempferol-3-*O*-rutinoside (**5**)	4.25±0.07#	4.55±0.19#*	4.75±0.26#*
Metformin	4.56±0.22#	5.31±0.23#*	6.06±0.15#*
Blank control [b]		6.48±0.36	
Insulin group		3.93±0.43#	

[a] Each value is presented as mean ± SD (n = 6); [b] Used as blank control
#p<0.05 vs. blank ; *p<0.05 vs. insulin.

Supplementary Figure HPLC chromatograms of the crude flavonoid extract (CFE) and crude ethanol extract (CEE). Experimental conditions: Waters Symmetry C_{18} column (5 μm, 4.6 mm× 250 mm, i.d.,); Mobile phase: acetonitrile/0.1% aqueous solution of acetic acid (16:84, *v/v*). Flow rate: 1.0 mL/min; Detection: 254 nm; Concentration: 20 mg/mL; Injection volume: 10 μL.

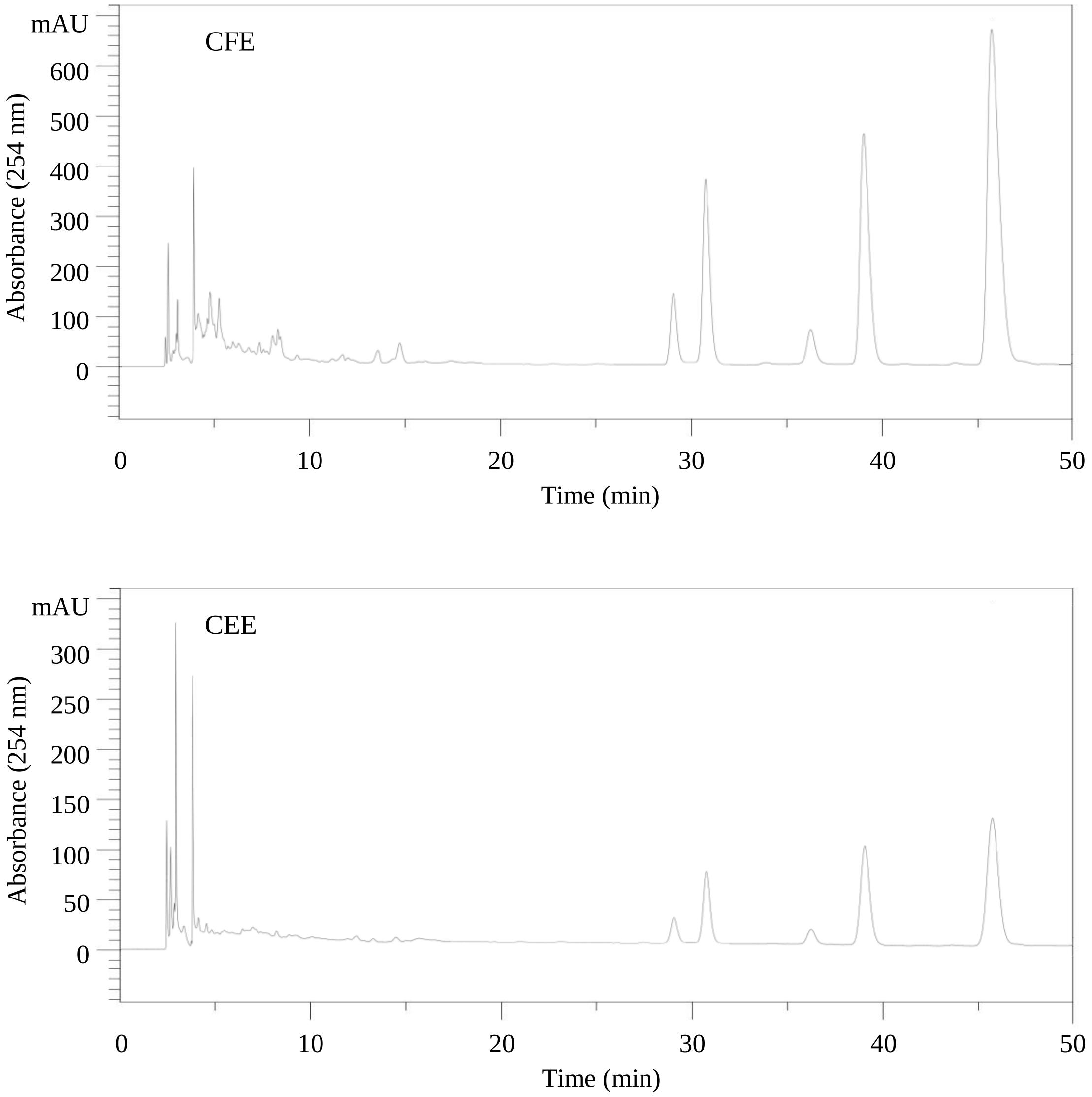

Supplement: Supplementary file 1 — Supplementary information. [file 41598_2020_65769_MOESM1_ESM.pdf]
